# Formaldehyde

**Published:** 1897-11

**Authors:** H. C. Wood

**Affiliations:** Philadelphia, Pa.


### Abstracts and Translations.

### FORMALDEHYDE.

BY H. C. WOOD, M.D., LL.D., PHILADELPHIA, PA.

The gaseous body known as formaldehyde, formyl, or formol, has been sufficiently studied in the laboratory to indicate that it is a very valuable addition to our practical medicaments. Owing to the efforts of an enterprising firm of pharmaceutical chemists it is chiefly known by the profession as formalin. Formalin is, however, an aqueous forty-per-cent. solution of formaldehyde, protected by a trade-mark name, and therefore loaded with all the excess of price, etc., which are of necessity associated with proprietary remedies.

We know of no reason, however, why doctor or patient should pay this tax, and as the demand grows, various solutions of formaldehyde will probably be put upon the market. Indeed, under the name of formaldehyde, Merck & Co. to-day sell an aqueous solution at the rate of seventy-five cents a pound, including the bottle; the corresponding price of formalin being eighty-five cents a pound, also including package; so that by purchasing the solution of Merck there is a saving of over twelve per cent. For the purposes of calculation and production of strength, a fifty-per-cent. solution would be preferable; but it appears not to be permanent, becoming after a time turbid.

As long ago as 1888, Trillat discovered the germicidal powers of formaldehyde, and in a tabulated statement of the results obtained by numerous observers, Marion, in 1895, showed there was a widely founded consensus of opinion that 1 part in 20,000 of the gas would kill most bacteria if the contact were prolonged. Most observers agree in stating that the germicidal power of formaldehyde is equivalent to that of corrosive sublimate; but later reports (as those of Walter, made in 1896) indicate that while it is many times stronger than carbolic acid, it is inferior in strength to corrosive sublimate, and that a contact of one hour is necessary for the destruction of *all pathogenetic spores* by its one-per-cent. solution. A very important observation is that of Aronson and Burkhard, according to whose experiments this substance is not merely germicidal, but has the power of stripping the toxins of diphtheria, of tetanus, and probably of other diseases of their poisonous powers.

The action of the drug upon the higher animals is comparatively feeble. Trillat states that sixty-six cubic centimetres of it per kilogramme are not fatal to the guinea-pig, although the urine passed by the poisoned animals is incapable of putrefaction; while no pronounced symptoms were produced in the rabbit by the injection of thirty-eight centigrammes per kilogramme. According to Mosso and Paoletti, fifty cubic centimetres per kilogramme injected hypodermically into the dog caused a progressive poisoning, with fall of temperature and death after many days. The same amount given by the stomach produced rapid effects, with violent convulsions, general rigidity, salivation, stupor, unconsciousness, and death. A curious fact was noted,—namely, that the drug was much more poisonous when taken by the stomach than when taken hypodermically. This is evidently connected with its intense irritant properties. It is probable that it causes a severe gastro-enteritis; and it is possible, especially as deep eschars have been noted following the hypodermic injections, that when it is injected into the cellular tissue the local effects may interfere with absorption. Small doses have been found by Mosso and Paoletti to increase the blood-pressure, probably by causing peripheral contraction of the arteries, while the large doses depress the circulation, and so act upon the blood that it coagulates with the separation of a dark-red serum.

The gas formaldehyde is exceedingly irritant, and it has been noted by Mosso and Paoletti that free inhalation is liable to produce severe pulmonary inflammation, ending, it may be, fatally.

Thus far formaldehyde has been used in practical medicine,—chiefly, first, as a preservative; second, as a germicide and disinfectant. In the anatomical laboratory it has been largely employed, and is, we believe, highly considered by most anatomists. Dr. Holmes, the university anatomical demonstrator, informs us, however, that a three-per-cent. solution will not keep bodies unless they are completely immersed in it, parts floating above the liquid rapidly drying and being destroyed by mould; also that the brain in its interior softens and putrefies in such solution. According to Professor Orth, the addition of a ten per cent. of a forty-per-cent. solution of formaldehyde to one hundred parts of Müller's solution greatly increases the preserving and hardening action of that solution. As, however, the compound solution begins to decompose in two days after its mixing it must be made freshly at the time of using.

The chief interest to the general profession, however, of formaldehyde centres at present in its powers as a germicide and disinfectant, and it looks as though it would, for all ordinary purposes of the hospital and sick-room, replace other substances. Hitherto there has been no practical method known of disinfecting apartments. A peculiarity of the gas formaldehyde is its power of penetrating not only animal tissues, but almost all organic substances, so that Van Ermengen and Sugg, in 1895, demonstrated in an elaborate series of experiments that it is possible to sterilize books by means of formaldehyde in an approximate quantity of five cubic centimetres to one litre of air; while in our own University Laboratory, Horton, in 1896, showed that books infected with various pathogenetic germs could be disinfected by being shut up for fifteen minutes in an atmosphere containing the vapor of commercial formalin, one cubic centimetre of the formalin to three hundred cubic centimetres or less of air. The method of Trillat, however, is without doubt greatly superior to evaporation or pulverization of the solution of formaldehyde.

It consists in the use of the formaldehyde directly after its production by the passage of the vapors of methylic alcohol over red-hot metal. Employing a very ingenious apparatus devised by himself, Trillat proved that it was possible to completely disinfect rooms and the furniture contained therein by the consumption during six hours of from four to six litres of methylic alcohol for each three hundred cubic metres of the room.

In the very recent (January, 1897) report of an elaborate series of experiments, Dr. J. J. Kinyoun, of the United States Marine Service, has confirmed all the statements of Trillat; and has further shown that none of the ordinary fabrics are injured by the gas, which is entirely capable of completely disinfecting curtains, carpets, clothing, bed-covering, and the minor forms of furniture, although it is doubtful whether heavy upholstered furniture, such as sofas and mattresses, can in their interior be thoroughly disinfected. Nevertheless, he did succeed in killing germs underneath ten layers of blankets.

There is at present offered for sale in the markets of the United States several forms of formaldehyde lamps, which, we believe, are all adaptations of the Trillat system, and are probably all of them efficient. We have known influenza apparently put an end to in a house where cases were continually recurring by its use, and the time seems not far distant when some such apparatus will be owned by every physician and used always in private practice where there has been germ-disease. The formaldehyde is so irritating that no one can stay in the room during the disinfection; but the apparatus is automatic and can be left to itself. Rooms can undoubtedly be disinfected by pulverizing in them with steam atomizers formalin or other solution of formaldehyde. There is, however, no reason for believing that the results obtained with formalin are better than those obtained with the gas formaldehyde; probably they are not as good. The expense with formalin is very many times greater than with formaldehyde. Thirty-two grammes of methylic alcohol theoretically should yield thirty grammes of liquid formaldehyde, as that compound exists under the influence of intense cold or under pressure. This amount of the liquid formaldehyde at ordinary pressure and a temperature of 60° F. should make 23.4 litres of the gas formaldehyde, the form in which the substance usually exists. Calculating this out, it will be found that one pound of methylic alcohol should yield three hundred and fifty-five litres of formaldehyde gas, or about four hundred quarts, or about one hundred gallons. As pure methylic alcohol is listed by Merck at one dollar and nine cents a pound, theoretically there should be obtained for one dollar and nine cents as much of the vapor as exists in two hundred and fifty gallons of formalin, a substance listed at eighty-five cents a pound. Even supposing that thirty per cent. of the methylic alcohol is wasted in the practical making of the formaldehyde, it is plain that the cost of the vapor is many times less than of a corresponding amount of formalin.

Formaldehyde has not as yet been fully tested in practical surgery, but there is sufficient knowledge to indicate that it may prove to be the best of the known germicides. The taste of it and the irritant quality of the solution, along with the feebleness of its toxic properties, make serious accidental poisoning by it very improbable; and also make still more improbable, practically impossible, any fatal results parallel to those which have been so frequently produced by the surgeon when by the too free use of corrosive sublimate or of carbolic acid he has caused the death of the unfortunate patient, a result, in our opinion, which has been far more frequent than is generally admitted.

It would appear also that formalin is not only a germicide, but will so act upon various organic materials as to remove bad odors; at least, according to Walter, a one-per-cent. solution will almost immediately and entirely deodorize fæces. For the cleansing of vessels in the anatomical laboratory the one-half-per-cent. solution of formaldehyde suffices, but for most purposes in the clinical amphitheatre the one-per-cent. solution may be used. According to Walter, washing the hands with it and afterwards with alcohol renders them completely antiseptic, while it does not stain and appears not to irritate more than the other antiseptic solutions used. It is stated that it does not in any way affect instruments, so that its use is superior to the method of boiling as commonly practised, because it leaves the edges of the knives undulled. We are also informed by surgeons that it affords a very efficient means of preparing catgut.

It seems to us probable that by the use of the gas itself many of the tedious procedures at present connected with heating in the preparation of surgical dressings may be avoided. All that would probably be necessary to do would be to connect the nozzle of a formaldehyde-forming lamp with the copper or tin vessel containing the dressings, so as to allow a current of the vapor to pass freely through in order to completely sterilize the dressing. Experiments are necessary, however, to show how far what seems probable *a priori* would endure in practice.

There appears to be little doubt as to the great value of formaldehyde as a local application, although its full powers have as yet been scarcely made out, while there appears to be no danger of poisoning by it, although its solution, if used too strong, is liable to produce excessive irritation of the sound tissues. In the University Hospital, as well as in the Presbyterian Hospital and in private practice, Professor Willard has used formaldehyde in all sorts of wounds, in carbuncles, and in various infected sores. For washing out and purifying an infected wound he employs a two-per-cent. solution. For a continuous local application, or for free irrigation, a quarter of the one-per-cent. solution; and while the effects upon suppuration and the general evidences of infection have been very pronounced, in no case has there been any local irritation. Professor von Winckel, of Berlin, reported to the gynæcological society of that city that he had found the agent extremely effective in gonorrhœal vaginitis and other infective diseases of the female genitals; and that while a four-per-cent. solution of formaldehyde could often be painted with great and immediate advantage upon the cervix or other diseased part, 1 part in 10,000 is effective by the method of irrigation. It is very probable that strong solutions of formaldehyde will be found to be a very valuable remedy in the treatment of chancroids, poisoned wounds, etc. Of course, these strong solutions should be used at first tentatively, and always as though they were mild caustics.

A suggestion, which we throw out for the testing of surgeons, is whether it be not possible by means of the simple formaldehyde-lamp to immediately disinfect wounds by allowing the vapors of the formaldehyde to be discharged for a few moments into the wound. The penetrating character of these vapors is such that *a priori*, at least, it appears probable that they would penetrate into the recesses of the wound much better than would a liquid solution. In bad cases of peritonitis, with the whole abdominal surfaces infected, it is notorious that laparotomy, although frequently practised, is almost invariably followed by death; but here again the possibilities of rapid disinfection and purification by means of the gaseous and seemingly scarcely toxic vapor appear to open out a possible avenue of safety. The momentary irritation of the peritoneum would probably not be severe enough to create serious shock; while our present knowledge indicates that it would interfere with rather than increase septic absorption.—*University Medical Magazine.*

